# Lipoprotein(a) as a cardiovascular risk factor among patients with and without diabetes Mellitus: the Mass General Brigham Lp(a) Registry

**DOI:** 10.1186/s12933-024-02348-2

**Published:** 2024-07-18

**Authors:** Arthur Shiyovich, Adam N. Berman, Stephanie A. Besser, David W. Biery, Rhanderson Cardoso, Sanjay Divakaran, Avinainder Singh, Daniel M. Huck, Brittany Weber, Jorge Plutzky, Christopher Cannon, Khurram Nasir, Marcelo F. Di Carli, James L. Januzzi, Deepak L. Bhatt, Ron Blankstein

**Affiliations:** 1 Division of Cardiovascular Medicine, Department of Medicine, Brigham and Women’s Hospital, Harvard Medical School, Boston, MA USA; 2 Department of Radiology, Brigham and Women’s Hospital, Harvard Medical School, Boston, MA USA; 3 Department of Cardiovascular Medicine, Division of Cardiovascular Prevention and Wellness, Houston Methodist DeBakey Heart and Vascular Center, Houston, TX USA; 4 Cardiology Division, Massachusetts General Hospital, Harvard Medical School, Baim Institute for Clinical Research, Boston, MA USA; 5 Mount Sinai Fuster Heart Hospital, Icahn School of Medicine at Mount Sinai, New York, NY USA

**Keywords:** Lipoprotein (a), Diabetes mellitus, Coronary artery disease outcomes

## Abstract

**Background:**

Diabetes mellitus (DM) and Lp(a) are well-established predictors of coronary artery disease (CAD) outcomes. However, their combined association remains poorly understood.

**Objective:**

To investigate the relationship between elevated Lp(a) and DM with CAD outcomes.

**Methods:**

Retrospective analysis of the MGB Lp(a) Registry involving patients ≥ 18 years who underwent Lp(a) measurements between 2000 and 2019. Exclusion criteria were severe kidney dysfunction, malignant neoplasms, and prior atherosclerotic cardiovascular disease (ASCVD). The primary outcome was a combination of cardiovascular death or myocardial infarction (MI). Elevated Lp(a) was defined as > 90th percentile (≥ 216 nmol/L).

**Results:**

Among 6,238 patients who met the eligibility criteria, the median age was 54, 45% were women, and 12% had DM. Patients with DM were older, more frequently male, and had a higher prevalence of additional cardiovascular risk factors. Over a median follow-up of 12.9 years, patients with either DM or elevated Lp(a) experienced higher rates of the primary outcome. Notably, those with elevated Lp(a) had a higher incidence of the primary outcome regardless of their DM status. The annual event rates were as follows: No-DM and Lp(a) < 90th% − 0.6%; No-DM and Lp(a) > 90th% − 1.3%; DM and Lp(a) < 90th% − 1.9%; DM and Lp(a) > 90th% − 4.7% (*p* < 0.001). After adjusting for confounders, elevated Lp(a) remained independently associated with the primary outcome among both patients with DM (HR = 2.66 [95%CI: 1.55–4.58], *p* < 0.001) and those without DM (HR = 2.01 [95%CI: 1.48–2.74], *p* < 0.001).

**Conclusions:**

Elevated Lp(a) constitutes an independent and incremental risk factor for CAD outcomes in patients with and without DM.

**Supplementary Information:**

The online version contains supplementary material available at 10.1186/s12933-024-02348-2.

## Introduction

Diabetes mellitus (DM) is a major cardiovascular risk factor and its global prevalence and associated burden continues to increase [1, 2]. Coronary artery disease (CAD) remains the leading cause of morbidity and mortality among patients with DM [3–5]. Lipoprotein(a) [Lp(a)] is a lipid-carrying particle that consists of a low-density lipoprotein (LDL)-like structure, incorporating apolipoprotein B-100 connected by a disulfide bond to apolipoprotein(a) [6]. Lp(a) was discovered over 60 years ago and has captured considerable interest in the cardiovascular field due to its established connection with atherosclerotic cardiovascular disease (ASCVD) in general and CAD in particular [7–9]. The recent introduction of potential therapies focused on lowering Lp(a) has brought new attention to this biomarker [6, 10].

Reports indicate that DM can impact lipid metabolism and contribute to the accelerated development of coronary atherosclerosis, a condition referred to as “diabetic dyslipidemia” [11, 12]. There are known variations in the distribution of Lp(a) levels among patients with DM, with some showing higher and others lower Lp(a) levels among individuals with DM [13–15]. Furthermore, studies suggest that although Lp(a) is associated with an increased risk of adverse cardiovascular events in patients with a history of atherosclerotic cardiovascular disease (ASCVD) (i.e. secondary prevention), the magnitude of this increased risk may vary between those with and without DM [16–19]. There is a deficiency in high-quality contemporary data evaluating the risk associated with elevated Lp(a) levels among individuals with and without DM, especially among those without a prior history of atherosclerotic cardiovascular disease (ASCVD). Therefore, the purpose of this study was to investigate the association between Lp(a) and CAD outcomes among patients with and without DM.

## Methods

### Study design and population

The patient population was derived from the Mass General Brigham Lp(a) Registry, as previously described [9, 20]. In brief, this retrospective cohort study included all individuals who underwent Lp(a) testing as part of their routine healthcare from January 2000 to July 2019. The research was carried out at two academic medical centers in Boston, Massachusetts: Brigham and Women’s Hospital and Massachusetts General Hospital.

### Ethical considerations

The Mass General Brigham Lp(a) Registry, encompassing the present study, received approval from the Institutional Review Board at Mass General Brigham. This study was conducted in accordance with the principles outlined in the Declaration of Helsinki. Informed consent waived due to retrospective analysis of anonymized data.

 All individuals aged 18 years or older with at least one Lp(a) result were screened for inclusion in the cohort. Exclusion criteria consisted of two factors: (1) severe kidney dysfunction, defined as stage 5 chronic kidney disease (estimated glomerular filtration rate < 15 mL/min/m2), prior renal transplant, or those undergoing renal replacement therapy; and (2) the presence of a diagnostic International Classification of Diseases (ICD) code for malignant neoplasm during the covariate assessment window, except for non-melanoma skin cancer. Moreover, for this study, individuals with a history of atherosclerotic cardiovascular disease (ASCVD), such as a previous myocardial infarction, a history of coronary revascularization, or a history of ischemic stroke, were also excluded.

### Data sources and definitions

The following sources were used to collect study data, including: (1) The Research Patient Data Registry (RPDR) [21] at Mass General Brigham, which provides demographic, laboratory, imaging, diagnostic, procedural, medication, vital status (based on the Social Security Administration Death Master File), and clinical documentation for individuals who meet specific search criteria. (2) ICD-coded death information from the National Death Index (NDI) and the Massachusetts Office of Vital Statistics was utilized to establish the causes of death for each patient who passed away during the study period.

To determine the presence of cardiovascular (CV) risk factors, validated natural language processing (NLP) modules [22], laboratory data, and diagnostic and procedural ICD-9, ICD-10, and Current Procedural Terminology (CPT) codes were utilized, as previously described [20]. The baseline covariate assessment period was established as the 12-month period before and 30 days after the Lp(a) measurement. For individuals with more than one Lp(a) test, their covariate assessment period was assessed relative to the first Lp(a) test.

Diabetes mellitus was defined by having at least two diagnostic ICD codes or two references via NLP during the covariate window. Alternatively, a single HbA1c > 6.5% test (for males of age or females > 50 years old) or two elevated HbA1c tests at least 400 days apart for females under the age of 50 to account for the possibility of gestational diabetes.

Non-Lp(a) dyslipidemia was determined through NLP, treatment with a cholesterol lowering medication, or laboratory values (median) that exceeded any one of the following thresholds during the covariate window: (1) total cholesterol ≥ 240 mg/dL, (2) LDL cholesterol ≥ 160 mg/dL, (3) HDL cholesterol < 40 mg/dL (men), (4) HDL cholesterol < 50 mg/dL (women), (5) total triglycerides ≥ 175 mg/dL.

### Lipoprotein(a) assays

Lp(a) testing occurred as part of routine medical care using either the Lp(a)-particle assay (measured in nmol/L) or the Lp(a)-mass assay (measured in mg/dL). All Lp(a) lab testing was conducted at commercial laboratories during the study period, utilizing industry-standard assays. To mitigate potential biases stemming from differences in Lp(a) testing techniques over the study period, percentile distributions were established separately for each assay, as previously described [9]. This approach was employed in prior large Lp(a) studies [23–26]. Considering the well-established distribution of Lp(a), predefined percentile groups were established and applied in this study: 1st-50th, 51st-70th, 71st-90th, and 91st-100th. After merging separate assays across percentiles, we then converted all Lp(a) values to nmol/L using the following conversion formula to best represent the data in a clinically relevant manner: Lp(a) nmol = (2.18 x Lp(a)-M) − 3.83 [25, 27]. Consequently, elevated Lp(a) was defined as a value surpassing the 90th percentile (≥ 216 nmol/L.)

### Primary outcome

The primary outcome was a combination of cardiovascular death or myocardial infarction (MI). As previously described [20], MI was defined by the presence of a diagnostic ICD code in the primary hospital discharge position. This methodology has been thoroughly validated and is associated with high specificity, high positive predictive value, and reasonable sensitivity [28–31]. Cardiovascular mortality was established using the ICD-coded underlying causes of death [32–34], as determined by the National Death Index (NDI) or the Massachusetts Office of Vital Statistics. The assessment of the cause of death was conducted blind to the Lp(a) levels or any other clinical factors.

### Statistical analysis

Baseline Characteristics were reported as median (interquartile range) for numerical characteristics and frequencies (percentages) for categorical variables. Baseline characteristics were analyzed as Mann-Whitney U tests for numerical characteristics and chi-square tests of association or Fisher exact tests for categorical variables. Patients were censored at either date of death or date of querying vital statistics. Patients without a cardiovascular death defined ICD code were censored at date of death and labeled as not experiencing cardiovascular death.

Log-rank tests were used to compare Kaplan-Meier Curves. Incident rate ratios and their 95% confidence intervals were reported for the primary outcome of cardiovascular death or acute myocardial infarction. Univariable and multivariable Cox proportional hazards regressions were analyzed separately for patients with and without DM to assess the association of Lp(a) percentile group with cardiovascular death or acute myocardial infarction. Separate individual outcomes were assessed as secondary outcomes. Cox proportional hazard regressions were reported as hazard ratios and 95% confidence intervals. Multivariable Cox regression models were assessed for multicollinearity using Spearman rank correlation and proportional hazard assumptions with Schoenfeld residuals, as well as being adjusted for the following covariates: age, sex, self-reported race and ethnicity, hypertension, non-Lp(a) dyslipidemia, and current smoking status. Wald test of coefficient was used to evaluate interaction term between Lp(a) and diabetes mellitus. Spline models were developed to assess a potential nonlinear relationship between continuous Lp(a) levels and the primary composite outcome in groups with and without DM. These models were constructed using 5 knots for each group.

All analyses were performed using Stata MP Version 18 (StataCorp, College Station, TX) and RStudio (version 2022.12.0) ggplot2 package (version 3.4.1). Two-tailed test with an alpha value of 0.05 were considered statistically significant.

## Results

A total of 6238 patients met the eligibility criteria, and among them the median age was 54 years, 45% were women, and 12% (733) had DM. The baseline characteristics of the study cohort and comparison between patients with and without DM are presented in Table 1. Patients with DM were older, more frequently male, and had a higher prevalence of other cardiovascular risk factors. There were no significant differences in the distribution of the Lp(a) values between patients with versus without DM (see Supplemental Fig. 1 for distribution). Furthermore, the median Lp(a) levels were similar between patients with and without DM, measuring 29 (IQR 12–99) versus 31 (IQR 11–107) nmol/L, respectively, *p* = 0.85.

**Table 1:** Baseline characteristics; patients with versus patients without diabetes mellitus

Baseline characteristic	All comers (*n* = 6,238)	Diabetes (*n* = 733)	No diabetes (*n* = 5505)	*p*-value
Demographics				
Age, median (IQR)	54 (43–64)	62 (52–71)	53 (42–64)	**< 0.001**
Female	2802 (44.9%)	302 (41.2%)	2500 (45.4%)	**0.031**
Race/Ethnicity, n (%)				**< 0.001**
White	5296 (84.9%)	596 (81.3%)	4700 (85.4%)	
Black	183 (2.9%)	41 (5.6%)	142 (2.6%)	
Hispanic	156 (2.5%)	26 (3.6%)	130 (2.4%)	
Asian	171 (2.7%)	17 (2.3%)	154 (2.8%)	
Other*	432 (6.9%)	53 (7.2%)	379 (6.9%)	
Past medical history, n (%)				
Hypertension	2054 (32.9%)	521 (71.1%)	1533 (27.9%)	**< 0.001**
Hyperlipidemia	3030 (48.6%)	529 (72.2%)	2501 (45.4%)	**< 0.001**
Diabetes type I	54 (0.9%)	54 (7.4%)	0 (0%)	**< 0.001**
Diabetes type II	679 (10.9%)	679 (92.6%)	0 (0%)	**< 0.001**
Chronic kidney disease	154 (2.5%)	63 (8.6%)	91 (1.7%)	**< 0.001**
Atrial fibrillation	363 (5.8%)	80 (10.9%)	283 (5.1%)	**< 0.001**
Heart failure	107 (1.7%)	39 (5.3%)	68 (1.2%)	**< 0.001**
Current smoker	1389 (22.3%)	192 (26.2%)	1197 (21.7%)	**0.007**
Former smoker	1570 (25.2%)	250 (34.1%)	1320 (24.0%)	**< 0.001**
Lab values, median (IQR)				
Hemoglobin A1C	5.6 (5.4–6.1); *n* = 3690	6.9 (6.2–7.6); *n* = 649	5.6 (5.3–5.8); *n* = 3041	**< 0.001**
Total Cholesterol, mg/dL	190 (163–221); *n* = 5047	174 (149–209); *n* = 633	192 (165–223); *n* = 4414	**< 0.001**
Triglycerides, mg/dL	112 (77 − 65); *n* = 4988	137.5 (90.5–203); *n* = 630	108 (76-159.5); *n* = 4358	**< 0.001**
LDL-C, mg/dL	109 (86–137); *n* = 4896	93.5 (73–111); *n* = 600	111 (89–138); *n* = 4296	**< 0.001**
HDL-C, mg/dL	51 (41–63); *n* = 5035	45 (37–54); *n* = 629	52 (42–65); *n* = 4406	**< 0.001**
Creatinine, mg/dL	1.0 (0.8–1.1); *n* = 4375	1.0 (0.8–1.2); *n* = 689	1.0 (0.8–1.1); *n* = 4,046	**< 0.001**
Median Lp(a), nmol/L	31.1 (11.4-107.4)	29.0 (12.0-98.6)	31.1 (11.4-107.4)	0.85
Medical therapy, n (%)				
Statins	2193 (35.2%)	461 (63.0%)	1732 (31.5%)	**< 0.001**
Non-statin lipid lowering therapies	295 (4.7%)	68 (9.3%)	227 (4.1%)	**< 0.001**
Insulin	198 (3.2%)	198 (27.0%)	0 (0%)	**< 0.001**
Non-Insulin diabetes therapies	317 (5.1%)	292 (39.8%)	25 (0.5%)	**< 0.001**

*Other includes Indian, Middle Eastern, Native American, Other, Pacific Islander, and unknown. Bold: *p* < 0.05

HDL-C: high-density lipoprotein cholesterol; Lp(a): lipoprotein a; LDL-C: low-density lipoprotein cholesterol

**Fig. 1:**
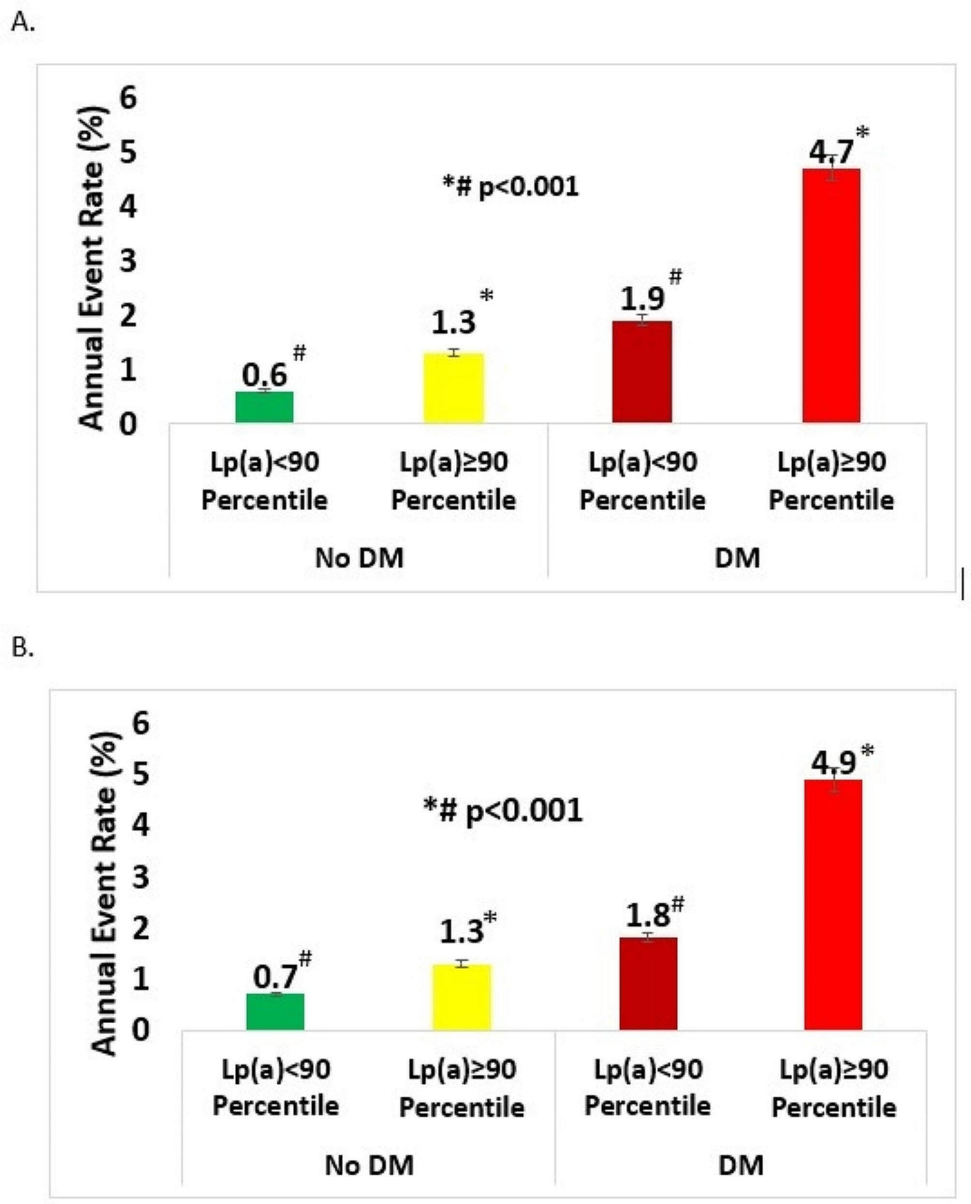
Unadjusted (**A**) and adjusted (**B**) annual event rates stratified by Lp(a) and DM status. Lp(a): Lipoprotein (a); DM: Diabetes mellitus. Adjusted for age, sex, hyperlipidemia, hypertension, smoking

**Fig. 2:**
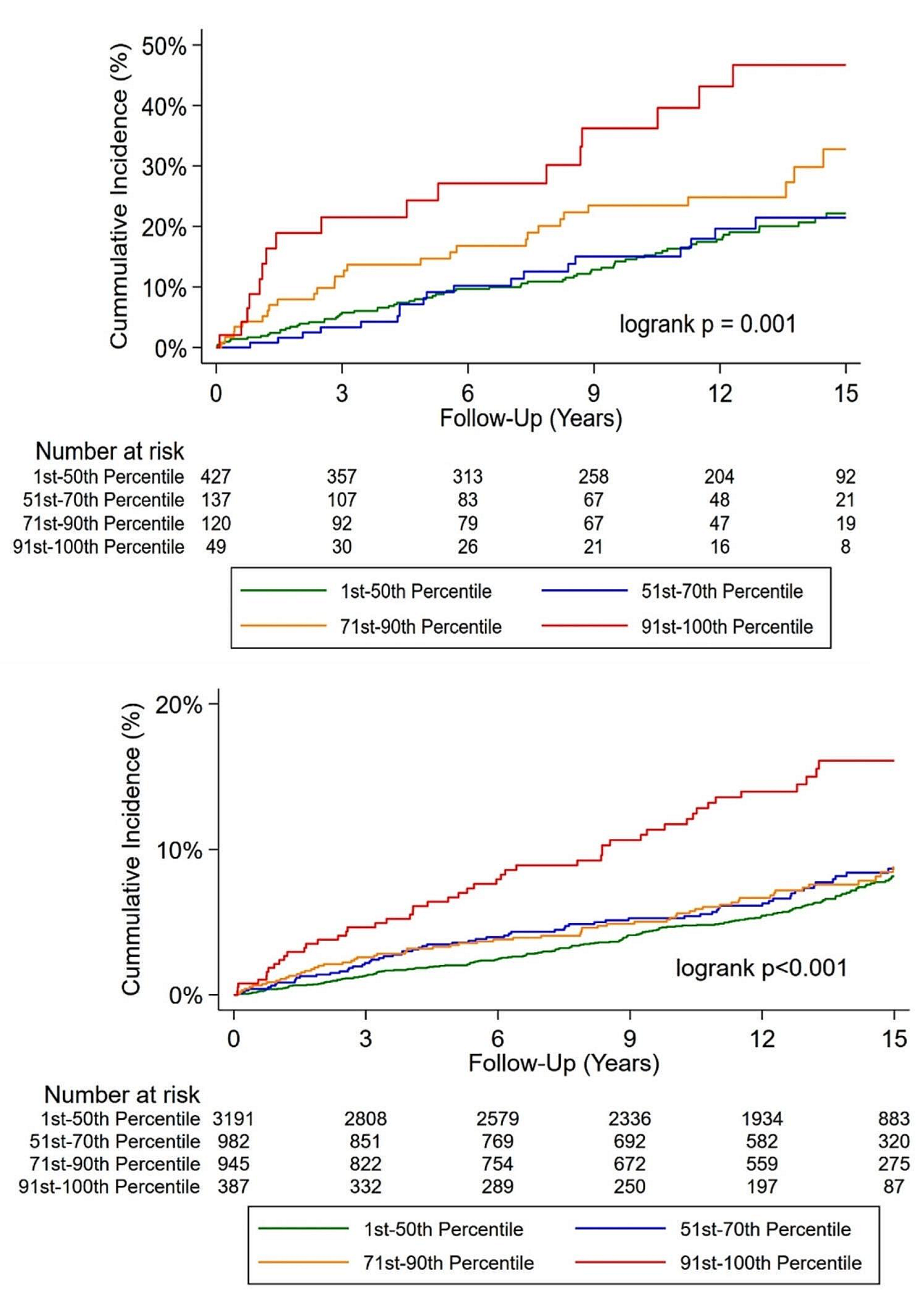
Cumulative incidence of the primary outcome (cardiovascular death or MI) among patients with (**A**) and without (**B**) diabetes mellitus stratified by Lp(a). Lp(a): Lipoprotein (a); MI: myocardial infarction

 Over a median follow-up period of 12.9 (IQR 7.7–15.3) years, individuals with either DM or elevated Lp(a) experienced higher rates of the primary outcome, as illustrated in Fig. 1. Notably, regardless of Lp(a) levels, patients with DM were found to have the highest annual event rates. For individuals with both DM and elevated Lp(a), the annual event rate of cardiovascular mortality or myocardial infarction (MI) was 4.7%, whereas the annual event rate was only 0.6% for those with neither condition. Findings were similar when annual event rates were adjusted for potential confounders. Notably, those with elevated Lp(a) had a higher incidence of the primary composite outcome regardless of DM status (Figs. 1 and 2).

When examining the association between the four pre-specified percentile subgroups of increasing Lp(a) with the primary outcome, patients with DM had a higher event rate among the group of 71-90th Lp(a) percentile (borderline statistical significance) when compared with the reference group (1-50th percentile). Among patients without DM, a higher event rate was only observed once when Lp(a) was greater than the 90th percentile. The restricted cubic spline analysis (Supplemental Fig. 2) demonstrated that higher Lp(a) levels were consistently associated with an increasing risk of the primary composite outcome for patients with and without DM. Notably among patients with DM the hazard ratio seemed to increase at a lower Lp(a) level.

Following adjustment for confounders (Table 2), elevated Lp(a) remained independently associated with the primary outcome in those with DM (HR = 2.66 [95%CI: 1.55–4.58], *p* < 0.001) and those without DM (HR = 2.01 [95%CI: 1.48–2.74], *p* < 0.001). There was no statistically significant interaction term between Lp(a) and DM (interaction *p* = 0.51).

**Table 2:** Unadjusted and adjusted hazard ratio (HR) for the primary outcome stratified by DM status and lp(a)

	Diabetes (*n* = 733)		No Diabetes (*n* = 5,505)	
	Unadjusted HR (95% CI)	Adjusted* HR (95% CI)*	Unadjusted HR (95% CI)	Adjusted* HR (95% CI)*
1st–50th percentile	Reference	–	Reference	–
51st–70th percentile	0.94 (0.57–1.53), *p* = 0.80	0.84 (0.51–1.38), *p* = 0.50	1.18 (0.90–1.54), *p* = 0.22	1.20 (0.92–1.57), *p* = 0.18
71st–90th percentile	1.48 (0.97–2.27), *p* = 0.072	1.23 (0.79–1.90), *p* = 0.36	1.14 (0.87–1.51), *p* = 0.33	1.09 (0.83–1.44), *p* = 0.53
91st–100th percentile	2.63 (1.55–4.46), *p* < 0.001	2.66 (1.55–4.58), *p* < 0.001	2.29 (1.69–3.11), *p* < 0.001	2.01 (1.48–2.74), *p* < 0.001

*Adjusted for age, sex, race, hypertension, hyperlipidemia, and current smoking

Similar findings were also found when examining the cumulative incidence of the individual outcomes of cardiovascular death or myocardial infarction stratified by Lp(a) levels and DM status (Supplemental Fig. 3). Specifically, regardless of DM status, patients with elevated Lp(a), had a markedly higher cumulative occurrence of each individual outcome. Following adjustment for confounders (Supplemental Table 1), elevated Lp(a) remained independently associated with each individual outcome in those with DM (cardiovascular Death: HR = 2.59 [95%CI: 1.38–4.84], *p* = 0.003, MI: HR = 3.79 [95%CI: 1.62–8.85], *p* = 0.002) and those without DM (CV Death: HR = 1.73 [95%CI: 1.19–2.51], *p* = 0.004, MI: HR = 3.21 [95%CI: 2.00–5.18], *p* < 0.001).

## Discussion

The present study, utilizing a comprehensive U.S. Lp(a) registry with long term follow-up, assessed the association of DM and Lp(a) levels with adverse cardiovascular events among individuals without a prior history of ASCVD. The main findings were as follows: (1) The incidence of the primary outcome of cardiovascular death or MI was elevated when either DM or elevated Lp(a) was present. However, the highest event rates were observed when both conditions were present. (2) Regardless of DM status, elevated Lp(a) was independently associated with the composite outcome of cardiovascular mortality or myocardial infarction (MI), as well as with each of the individual components. (3) Regardless of Lp(a) levels, patients with DM had significant higher event rates than those without DM.

These results are consistent with earlier studies, predominantly centered on patients with DM in secondary prevention studies, that indicated an association between elevated Lp(a) levels and an increased incidence of adverse cardiovascular outcomes [17–19].

The distribution of Lp(a) observed in the current study, particularly the 90th percentile threshold, is comparable to those reported in other observational registries (where the 90th percentile ranges between 43.5 and 75 mg/dL) [35–37]. However, this comparison may be limited by the variability in assays, laboratories, and conversion methods.

Our findings are also consistent with a large European study by Waldeyer et al. [36] revealing a higher coronary and cardiovascular risk among individuals with DM and elevated Lp(a). Notably, there are some key differences between our study and the European study. The prevalence of diabetes mellitus was significantly higher in our population (12% vs. 5.4%), likely reflecting recent trends of obesity and diabetes in the US. Additionally, the rate of lipid-lowering treatments, particularly statins, was significantly higher in our cohort, possibly indicating a more contemporary practice. We also evaluated risk associated with different Lp(a) thresholds among patients with and without diabetes separately, and conducted univariate and multivariable analyses of outcome by various Lp(a) values in these populations.

Nonetheless, a recent investigation conducted by Li et al. [16], which involved patients experiencing ST-elevation myocardial infarction (STEMI) and undergoing emergency revascularization, unveiled a distinct association between Lp(a) levels and the risk of adverse cardiovascular outcomes among individuals with and without DM. Specifically, elevated Lp(a) levels were linked to an increased risk in patients with DM, but not in those without DM. The variation in their findings compared to our study might be attributed to the distinction between a secondary prevention population in their cohort versus a primary prevention cohort in our study. Other factors that may contribute to these differences involve variations in Lp(a) thresholds within the Chinese population compared to American populations, a majority of male patients versus a more evenly distributed population in our study, and potential shifts in Lp(a) levels following myocardial infarction [16, 38, 39]. A recent study by Yu et al. [13] explored the threshold value of Lp(a) for the development of coronary artery disease (CAD) in individuals with suspected CAD, both with and without DM, and examined the impact of Lp(a) on CAD at optimal LDL-C levels. Consistent with our findings the authors reported that elevated Lp(a) was an independent risk factor for CAD in patients with or without DM. In addition, they found that Lp(a) had a different threshold value for the occurrence of CAD in patients with and without DM. As also supported by our findings, they reported that the threshold value of Lp(a) for the risk of CAD in DM patients was lower, than for those without DM. Our study aligned with their results concerning the heightened risk of adverse cardiovascular outcomes in patients with DM compared to those without. Additionally, we noted a higher incidence of the primary outcome among patients with DM in the 71-90th Lp(a) percentile range, which was not evident among patients without DM.

Several mechanisms could play a role in differential impact of Lp(a) between patients with versus without DM as following: first, individuals with DM are more likely to coronary atherosclerosis, even in the absence of prior ASCVD events, Thus, it is plausible that elevated Lp(a) may demonstrate a higher propensity for atherothrombotic events once coronary artery disease (CAD) is established. For example, findings from the MESA study indicate that when coronary artery calcium (CAC) is zero, there is no surplus risk associated with elevated Lp(a). However, in the presence of significant coronary atherosclerosis (CAC > 100), the risk associated with elevated Lp(a) becomes more robust [40]. Second, there could be a potential association between Lp(a) levels and the incidence of DM, although this was not consistently shown in the literature [13, 14, 41, 42] and not observed in our study. Third, a higher event rate associated with higher Lp(a) in diabetes could be due to additional confounding effects such as the presence of diabetic dyslipidemia [11, 12] which refers to negative effects of DM over lipid metabolism which contribute to the accelerated development of coronary atherosclerosis. Fourth, elevated levels of Lp(a) could potentially indicate both insulin resistance and pro-inflammation [43, 44]. Fifth, there could be a potentially synergistic effect between Lp(a) and high glucose levels, leading to damage to the vascular endothelium, increased susceptibility to vascular complications, and ultimately a heightened vulnerability to adverse cardiovascular events. Although patients with diabetes mellitus (DM) had higher hazard ratios (HR) compared with those without DM, we did not find a significant interaction between DM and lipoprotein(a) [Lp(a)], which aligns with the findings of by Waldeyer et al. [36]. However, our methodology may have been insufficient to detect such interactions due to limitations in sample size, and the fact that we evaluated a primary prevention population over a long-term follow-up.

Our findings advocate for the routine inclusion of Lp(a) testing for risk stratification and consideration of preventive pharmacotherapies particularly among individuals with DM. Furthermore, future trials exploring treatments aimed at lowering Lp(a) should consider focusing on patients with DM, particularly given the ongoing efforts to identify high-risk “primary” prevention cohorts.

## Limitations

The current study has certain limitations that should be acknowledged. First, this study is based on a retrospective registry from a single geographic region of the US, which could limit the generalizability of the findings particularly as our patient population was predominantly white. Second, the evaluation of DM and the other risk factors was conducted retrospectively around the time of Lp(a) measurement, which was a priori defined as the baseline covariate window. However, this could result in some underestimation as patients may develop additional risk factors over additional follow-up time over time. Third, Lp(a) testing was conducted as a routine part of clinical care, with the specific indications for testing unknown, thus potentially introducing selection bias. Nonetheless, our focus was on patients without a history of ASCVD who were referred for testing. Therefore, these findings remain generalizable to the population of patients in the U.S. for whom Lp(a) is presently tested, as routine testing for Lp(a) in patients without ASCVD is not commonly performed. Fourth, Lp(a) was measured using various techniques within our cohort, leading to potential variations, as there are limitations when converting between different units/measurements. Nevertheless, we standardized and assessed Lp(a) levels across assays using percentile distributions, consistent with the approach adopted by numerous previous studies. Fifth since we lacked reliable laboratory data on proteinuria range, this variable was not used for excluding patients, which may have led to biased Lp(a) values in a small subset of patients. However, some patients with proteinuria may be accounted for under the CKD exclusion criteria. Therefore, the likelihood of this biasing our results is low. Other potential confounders such as family history of ASCVD and C-reactive protein were not accounted for. Sixth, due to limited power, we were not able to delineate differences across various thresholds and cardiovascular events between subgroups of patients.

## Conclusions

Among individuals without a history ASCVD, there was a higher incidence of cardiovascular death or myocardial infarction when either DM or elevated Lp(a) was present, reaching a particularly high level when both conditions coexisted. While patients with DM had a considerably higher event rate than those without DM, Lp(a) was a robust risk marker among patients both with and without DM. Notably, the threshold of Lp(a) associated with higher risk was lower among individuals with DM. Our findings support the use of Lp(a) testing stratification among individuals with DM.

### Electronic supplementary material

Below is the link to the electronic supplementary material.

Supplementary Material 1

## Data Availability

No datasets were generated or analysed during the current study.

## Abbreviations

AMI: Acute myocardial infarction
ASCVD: Atherosclerotic cardiovascular disease
CAD: Coronary artery disease
DM: Diabetes Mellitus
ICD: International Classification of Diseases (ICD)
Lp(a): Lipoprotein (a)
LDL: Low density lipoprotein
MI: myocardial infarction
NLP: Natural language processing
