# Supplementary material for: Lipoprotein(a) as a cardiovascular risk factor among patients with and without diabetes Mellitus: the Mass General Brigham Lp(a) Registry
Source: Cardiovasc Diabetol. 2024 Jul 18;23:257. doi: 10.1186/s12933-024-02348-2 (PMC11264681; doi:10.1186/s12933-024-02348-2)
Supplement: Supplementary file 1 — Supplementary Material 1 [file 12933_2024_2348_MOESM1_ESM.docx]

**Lipoprotein(a) as a Cardiovascular Risk Factor Among Patients With and Without Diabetes Mellitus:** **The Mass General Brigham Lp(a) Registry**

**Supplemental Material**

**Table of Contents**

|  | Supplemental Figure 1 | 2 |
| --- | --- | --- |
|  | Supplemental Figure 2  Supplemental Figure 3  Supplemental Table 1 | 3  4  5 |
|  |  |  |
|  |  |  |
|  |  |  |

**Supplemental Figure 1.** Lp(a) distribution among patients with and without diabetes mellitus

| A) Patients with Diabetes  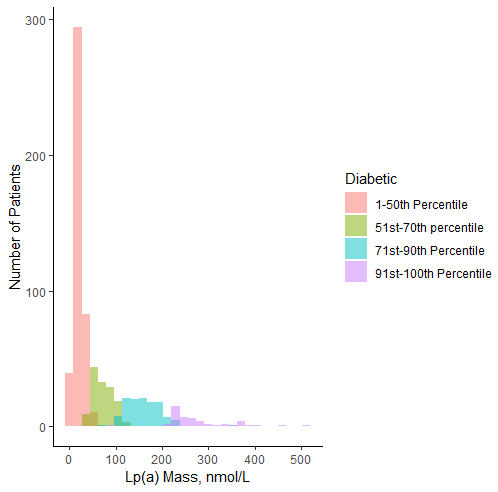 | B) Patients without Diabetes  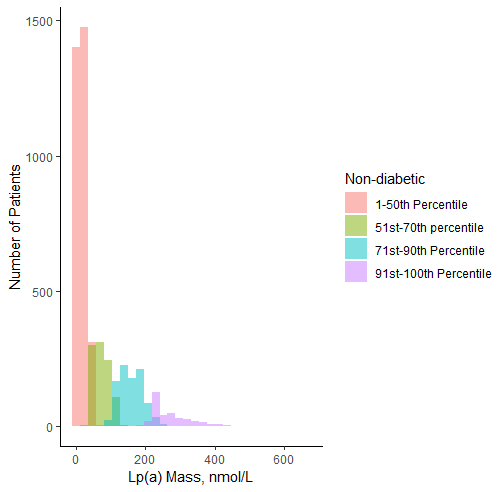 |
| --- | --- |

|  | **All Comers** | **Diabetes (n=733)** | **No Diabetes** | **p** |
| --- | --- | --- | --- | --- |
| **Lp(a) Percentiles** |  |  |  | .64 |
| 1^st^ -25^th^ Percentile | 1,922 (30.8%) | 216 (29.5%) | 1,706 (31.0%) |  |
| 26^th^-50^th^ Percentile | 1,611 (25.8%) | 199 (27.2%) | 1,412 (25.7%) |  |
| 51^st^-70^th^ Percentile | 1,194 (19.1%) | 150 (20.5%) | 1,044 (19.0%) |  |
| 71^st^-90^th^ Percentile | 1,045 (16.8%) | 114 (15.6%) | 931 (16.9%) |  |
| 91^st^-100^th^ Percentile | 466 (7.5%) | 54 (7.4%) | 412 (7.5%) |  |

**Supplemental Figure 2.** The association of Lp(a) with the primary outcome in patients with (A) and without (B) DM

A.


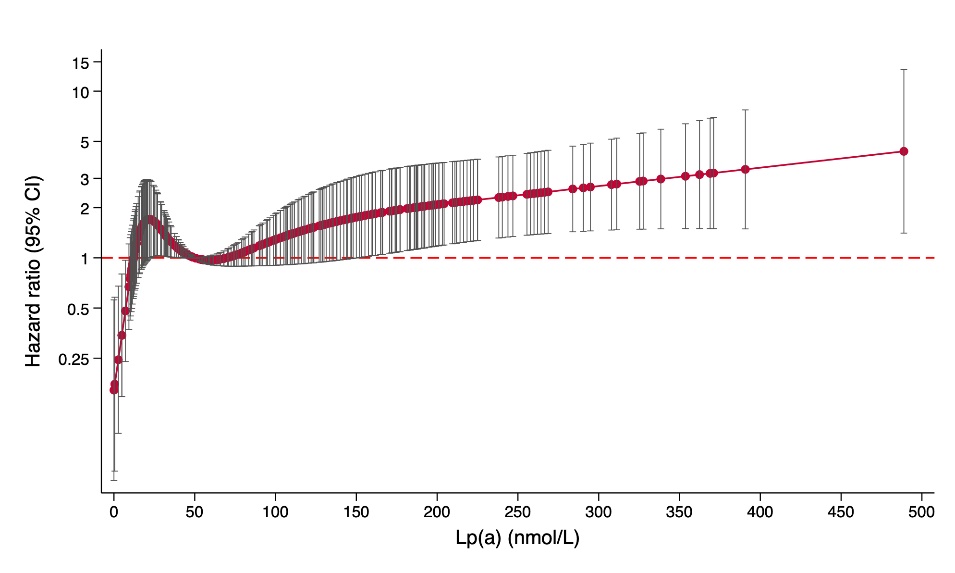


B

**
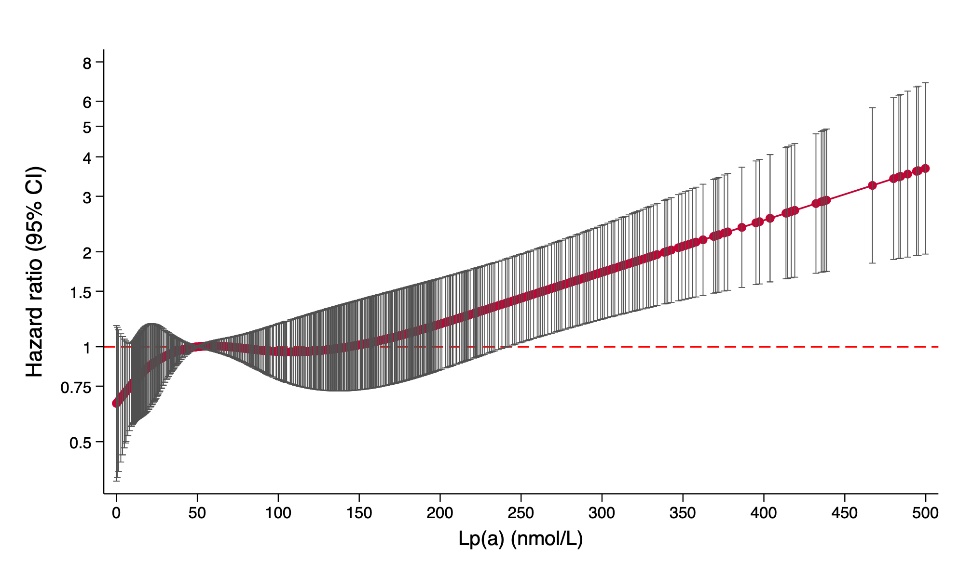
**

**Supplemental Figure 3.** Cumulative incidence of individual outcomes stratified by Lp(a) levels and diabetes mellitus status

| Cardiovascular Death | |
| --- | --- |
| 1. Patients with diabetes mellitus   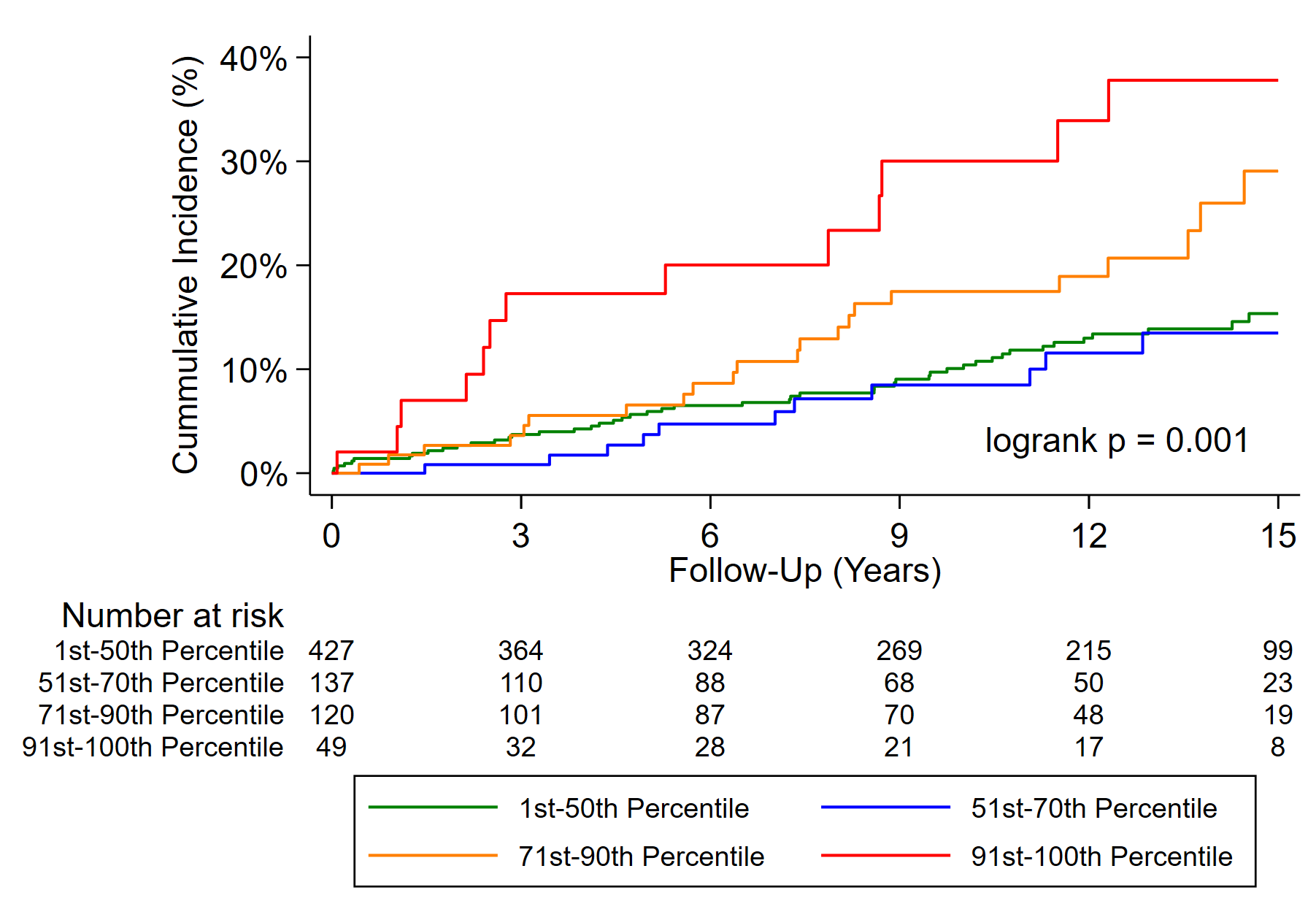 | 1. Patients without diabetes mellitus   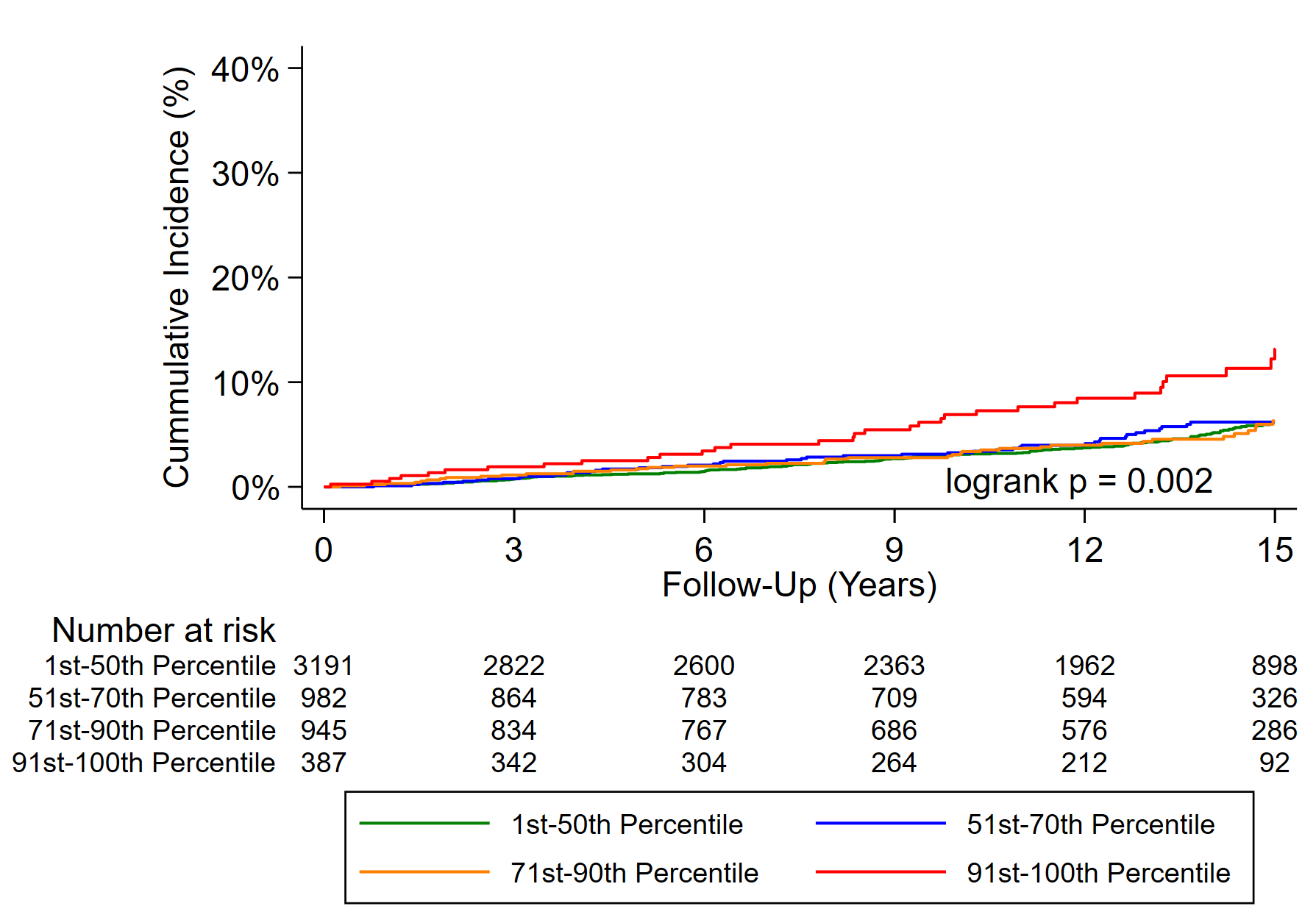 |
| Myocardial Infarction | |
| 1. Patients with diabetes mellitus   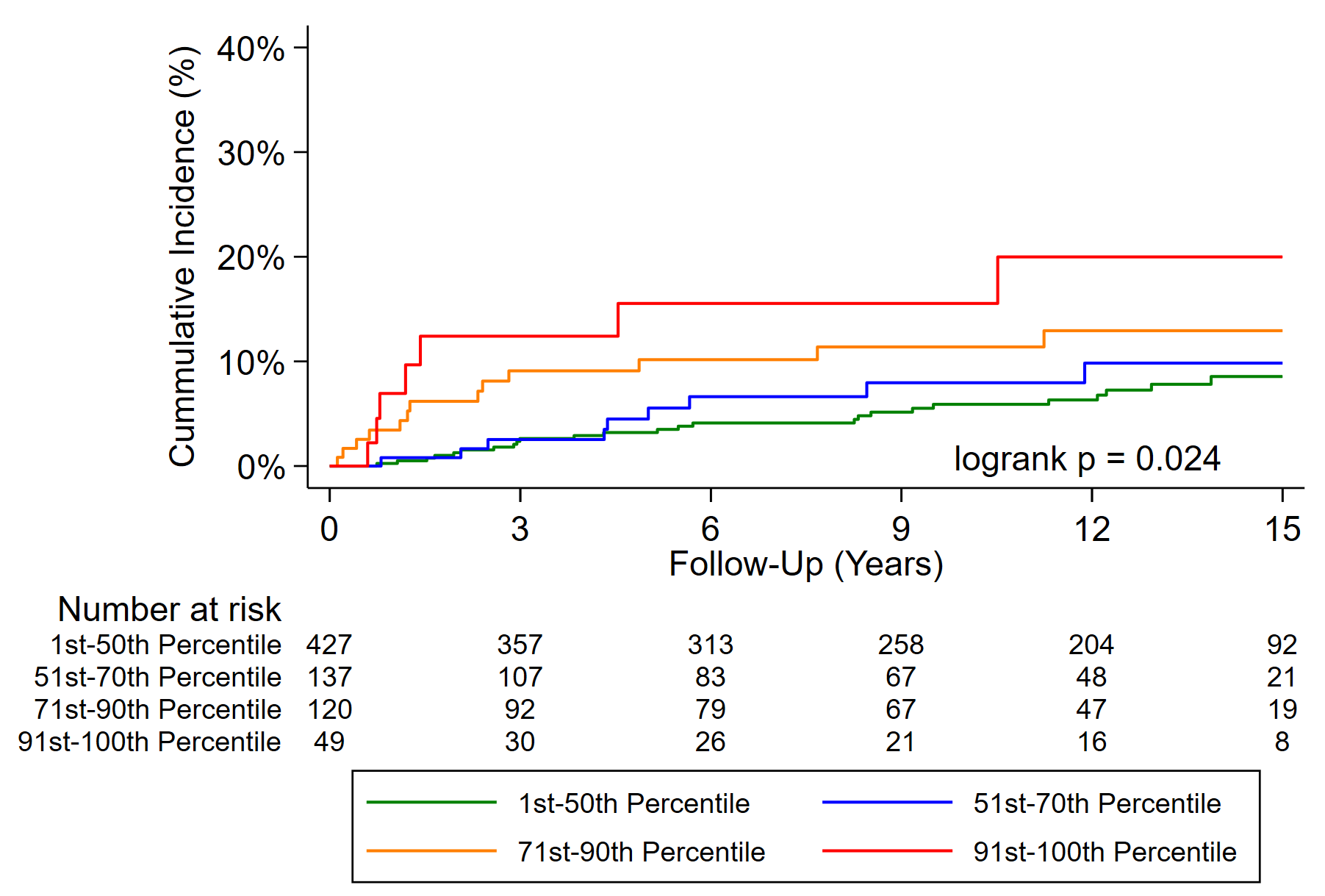 | 1. Patients without diabetes mellitus   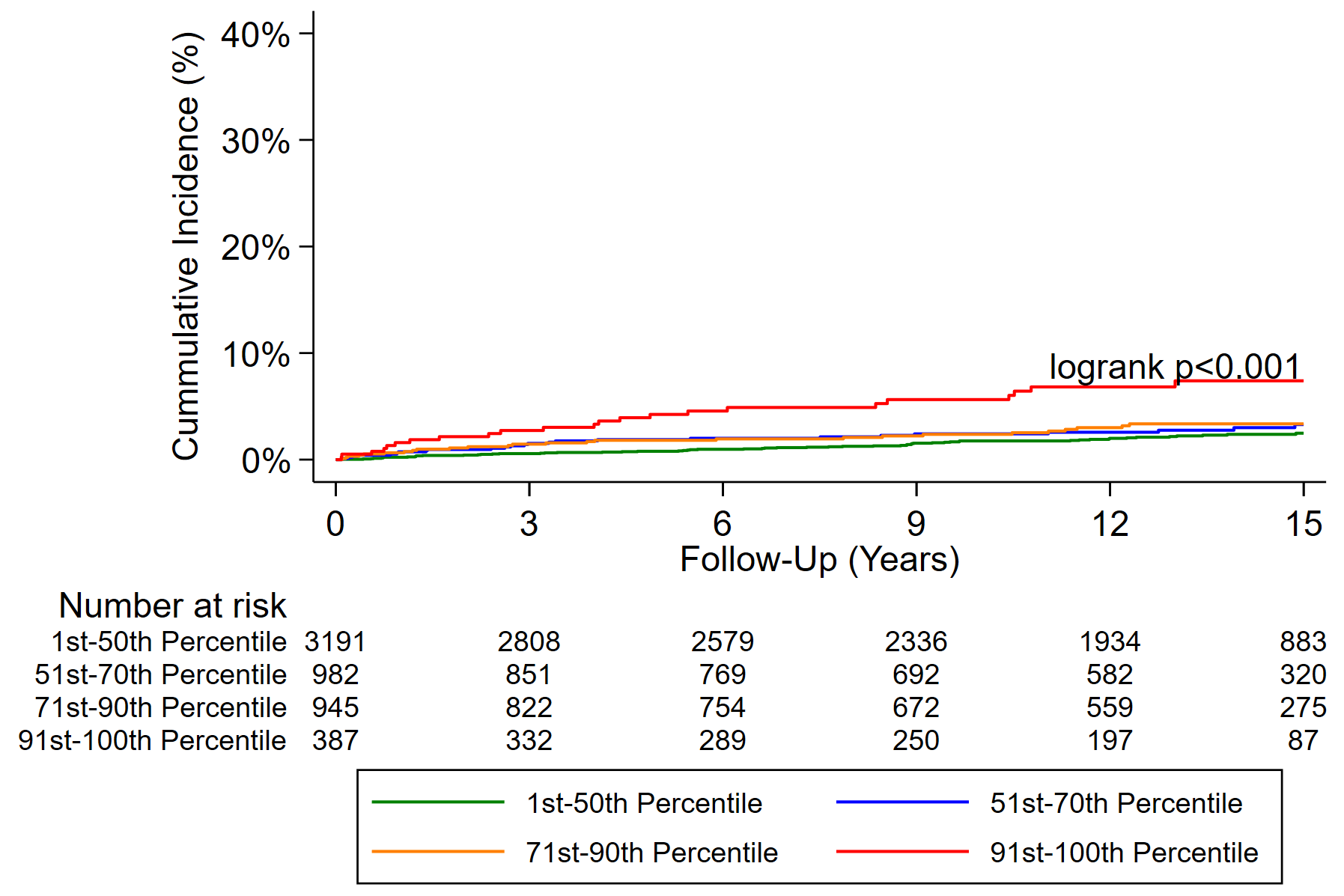 |

**Supplemental Table 1.** Unadjusted and adjusted Hazard Ratio (HR) for the primary outcome stratified by DM status and Lp(a)

|  | **Cardiovascular Death** | | | | **Myocardial Infarction** | | | |
| --- | --- | --- | --- | --- | --- | --- | --- | --- |
|  | **Patients with Diabetes (n= 733)** | | **Patients without Diabetes (n=5,505)** | | **Patients with Diabetes (n= 733)** | | **Patients without Diabetes (n=5,505)** | |
|  | **Unadjusted HR (95% CI)** | **Adjusted* HR (95% CI) *** | **Unadjusted HR (95% CI)** | **Adjusted* HR (95% CI)*** | **Unadjusted HR (95% CI)** | **Adjusted* HR (95% CI) *** | **Unadjusted HR (95% CI)** | **Adjusted* HR (95% CI)*** |
| 1^st^-50^th^ Percentile | Reference | -- | Reference | -- | Reference | -- | Reference | -- |
| 51^st^-70^th^ Percentile | 0.79 (0.43-1.48), p=0.469 | 0.68 (0.36-1.27), p=0.228 | 1.12 (0.82-1.53), p=0.479 | 1.16 (0.84-1.58), p=0.367 | 1.21 (0.57-2.58), p=0.623 | 1.19 (0.55-2.56), p=0.653 | 1.37 (0.87-2.16), p=0.180 | 1.41 (0.89-2.23), p=0.144 |
| 71^st^-90^th^ Percentile | 1.61 (0.99-2.62), p=0.055 | 1.17 (0.71-1.94), p=0.536 | 1.03 (0.74-1.42), p=0.878 | 0.96 (0.69-1.33), p = 0.798 | 1.92 (0.99-3.74), p=0.055 | 2.05 (1.04-4.04), **p=0.039** | 1.53 (0.98-2.39), p=0.063 | 1.47 (0.94-2.30), p=0.091 |
| 91^st^-100^th^ Percentile | 2.79 (1.52-5.11), **p=0.001** | 2.59 (1.38-4.84), **p=0.003** | 2.03 (1.40-2.93), **p<0.001** | 1.73 (1.19-2.51), **p=0.004** | 3.09 (1.34-7.13), **p=0.008** | 3.79 (1.62-8.85), **p=0.002** | 3.40 (2.12-5.44), **p<0.001** | 3.21 (2.00-5.18), **p<0.001** |

*****Adjusted for age, sex, race, hypertension, hyperlipidemia, and current smoking
